# A guide to selecting high-performing antibodies for Prosaposin (UniProt ID: P07602) for use in western blot and immunoprecipitation

**DOI:** 10.12688/f1000research.177779.1

**Published:** 2026-07-04

**Authors:** Vera Ruíz Moleón, Sara González Bolívar Bolívar, Riham Ayoubi, Vincent Francis, Peter S. McPherson, Carl Laflamme

**Affiliations:** 1 Department of Neurology and Neurosurgery, Structural Genomics Consortium, The Montreal Neurological Institute,, McGill University, Montreal, Québec, H3A 2B4, Canada

**Keywords:** P07602, PSAP, Prosaposin, Sphingolipid activator protein 1 (SAP-1), antibody characterization, antibody validation, western blot, immunoprecipitation, immunofluorescence

## Abstract

Prosaposin functions as a regulator of lysosomal enzymatic activity and as a neuroprotective player when secreted. Here we have characterized nine Prosaposin commercial antibodies for western blot and immunoprecipitation using a standardized experimental protocol based on comparing read-outs in knockout cell lines and isogenic parental controls. These studies are part of a larger, collaborative initiative seeking to address antibody reproducibility issues by characterizing commercially available antibodies for human proteins and publishing the results openly as a resource for the scientific community. While the use of antibodies and protocols vary between laboratories, we encourage readers to use this report as a guide to select the most appropriate antibodies for their specific needs.

## Introduction

Prosaposin, encoded by the *PSAP* gene, is a 527-amino acid protein, 70 kDa glycoprotein that serves as the precursor for four active saposins (A, B, C, and D). Following proteolytic cleavage, each saposin proteins plays a distinct role in the degradation of lysosomal sphingolipids. ^1^ In its full-length form, Prosaposin is secreted ^2^ and acts as a neurotrophic factor with protective effects. ^3^ Loss of Prosaposin function results in a phenotype resembling type 2 Gaucher’s disease, an acute neuropathic disorder. ^4^ Additionally, variants in the saposin D domain of *PSAP* gene have been directly linked to Parkinson’s disease. ^5^

This research is part of a broader collaborative initiative in which academics, funders and commercial antibody manufacturers are working together to address antibody reproducibility issues by characterizing commercial antibodies for human proteins using standardized protocols, and openly sharing the data. ^6^ It consists of identifying human cell lines with adequate target protein expression and the development/contribution of equivalent knockout (KO) cell lines, followed by antibody characterization procedures using most commercially available antibodies against the corresponding protein. ^6^ Here we characterized nine commercial Prosaposin antibodies, selected and donated by participant antibody manufacturers, for use in western blot and immunoprecipitation, enabling biochemical and cellular assessment of Prosaposin properties and function.

The authors do not engage in result analysis or offer explicit antibody recommendations. Our primary aim is to deliver top-tier data to the scientific community, grounded in Open Science principles. This empowers experts to interpret the characterization data independently, enabling them to make informed choices regarding the most suitable antibodies for their specific experimental needs. Guidelines on how to interpret antibody characterization data found in this study are featured on the YCharOS gateway ^7^ and in Table 4 of this data note. ^6^ Guidelines on how to interpret antibody characterization data found in this study are featured on the YCharOS gateway ^7^ and in Table 4 of this data note. ^6^

**Table 1.** Summary of the cell lines used.

Institution	Catalog number	RRID (Cellosaurus)	Cell line	Genotype
Abcam	ab255928	CVCL_0030	HeLa	WT
Abcam	ab265989	CVCL_B2CB	HeLa	*PSAP* KO

**Table 2.** Summary of the Prosaposin antibodies tested.

Company	Catalog number	Lot number	RRID (Antibody Registry)	Clonality	Clone ID	Host	Concentration (μg/μL)	Vendors recommended applications
Abcam	ab166910 **	1074892–2	AB_3105949	recombinant mono	EPR10784(B)	rabbit	0.21	Wb
Abcam	ab308122 **	1044141–3	AB_3101782	recombinant mono	EPR25649–20	rabbit	0.51	Wb, IP, IF
Bio-Techne (Novus Biologicals)	NBP2–37422 *	140523	AB_3096985	monoclonal	4D5F4	mouse	1.00	Wb, IF
Bio-Techne (R&D Systems)	AF8520	CJCW0123121	AB_3096981	polyclonal	-	rabbit	1.05	Wb, IF
GeneTex	GTX101064	44433	AB_2037779	polyclonal	-	rabbit	1.98	Wb
Proteintech	10801–1-AP	00116626	AB_2172462	polyclonal	-	rabbit	0.70	Wb, IF
Thermo Fisher Scientific	MA5–17159 *	ZF4351939	AB_2538630	monoclonal	4D5F4	mouse	1.00	Wb, IF
Thermo Fisher Scientific	MA5–38610 *	ZF4357167	AB_2898522	monoclonal	3B4A8	mouse	1.00	Wb, IF
Thermo Fisher Scientific	MA5–48579 *	ZF4351933B	AB_3093481	monoclonal	3A5H7	mouse	1.00	Wb

Wb = western blot; IP = immunoprecipitation, IF = immunofluorescence,

** recombinant antibody,

* monoclonal antibody

**Table 3.** Table of secondary antibodies used.

Company	Secondary antibody	Catalog number	RRID (Antibody Registry)	Clonality	Concentration (μg/μL)	Working concentration (μg/mL)
Thermo Fisher Scientific	HRP-Goat Anti-Rabbit Antibody (H + L)	65–6120	AB_2533967	polyclonal	1.0	0.2
Thermo Fisher Scientific	HRP-Goat Anti-Mouse Antibody (H + L)	62–6520	AB_2533947	polyclonal	1.5	0.75
Cell Signaling Technology	Protein A, HRP conjugate	12291	NA	polyclonal	0.125	0.5

**Table 4.** Illustrations to assess antibody performance in all western blot, immunoprecipitation and immunofluorescence.

Western blot	Immunoprecipitation
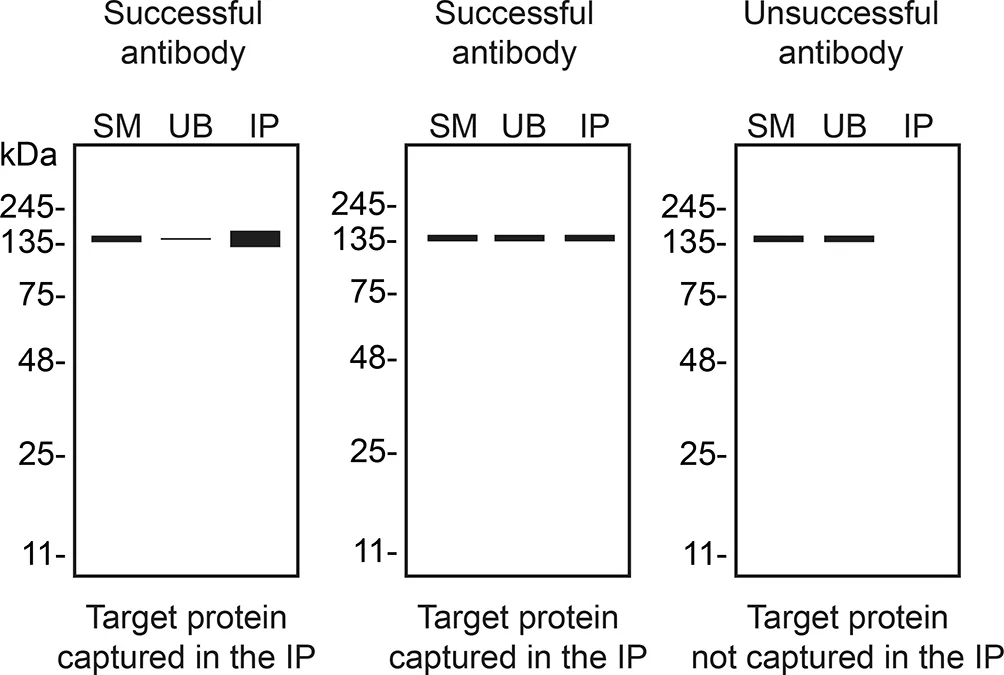	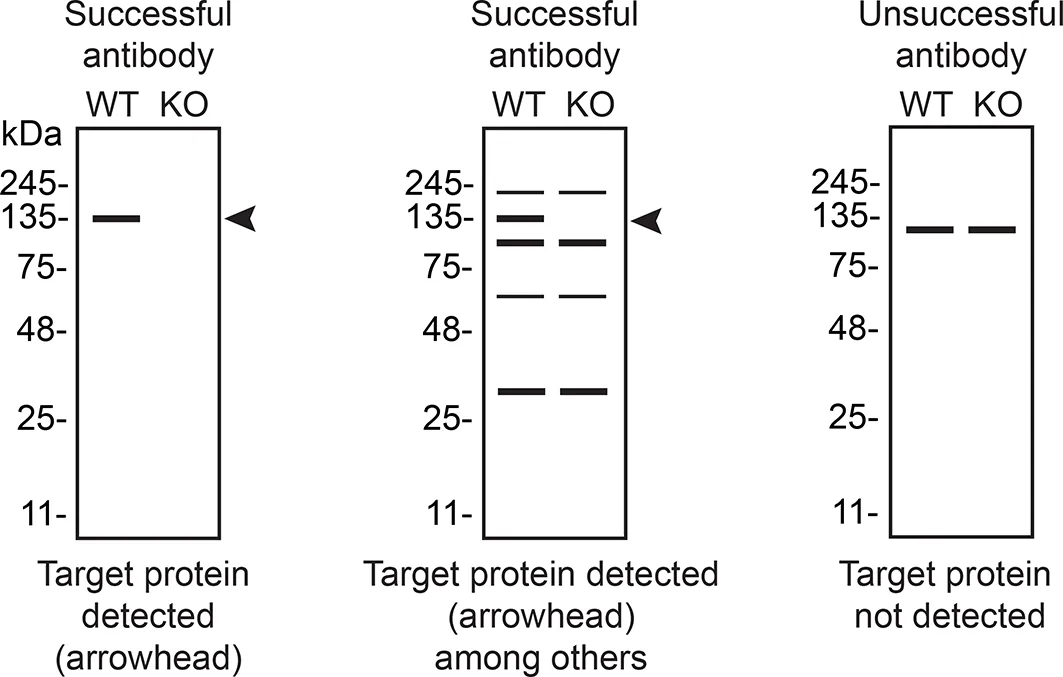

## Results and discussion

Our standard protocol involves comparing readouts from wild type (WT) and KO cell lines. ^8^ ^,^ ^9^ The first step was to identify a cell line(s) that expresses sufficient levels of a given protein to generate a measurable signal using antibodies. To this end, we examined the DepMap (Cancer Dependency Map Portal, RRID: SCR_017655) transcriptomics database to identify all cell lines that express the target at levels greater than 2.5 log _2_ (transcripts per million “TPM” + 1), which we have found to be a suitable cut-off. ^10^ HeLa expresses the *PSAP* transcript at 8.4 log _2_ TPM + 1. A *PSAP* KO cell line in HeLa was obtained from Abcam and was used for this study ( Table 1).

**Figure 1.**
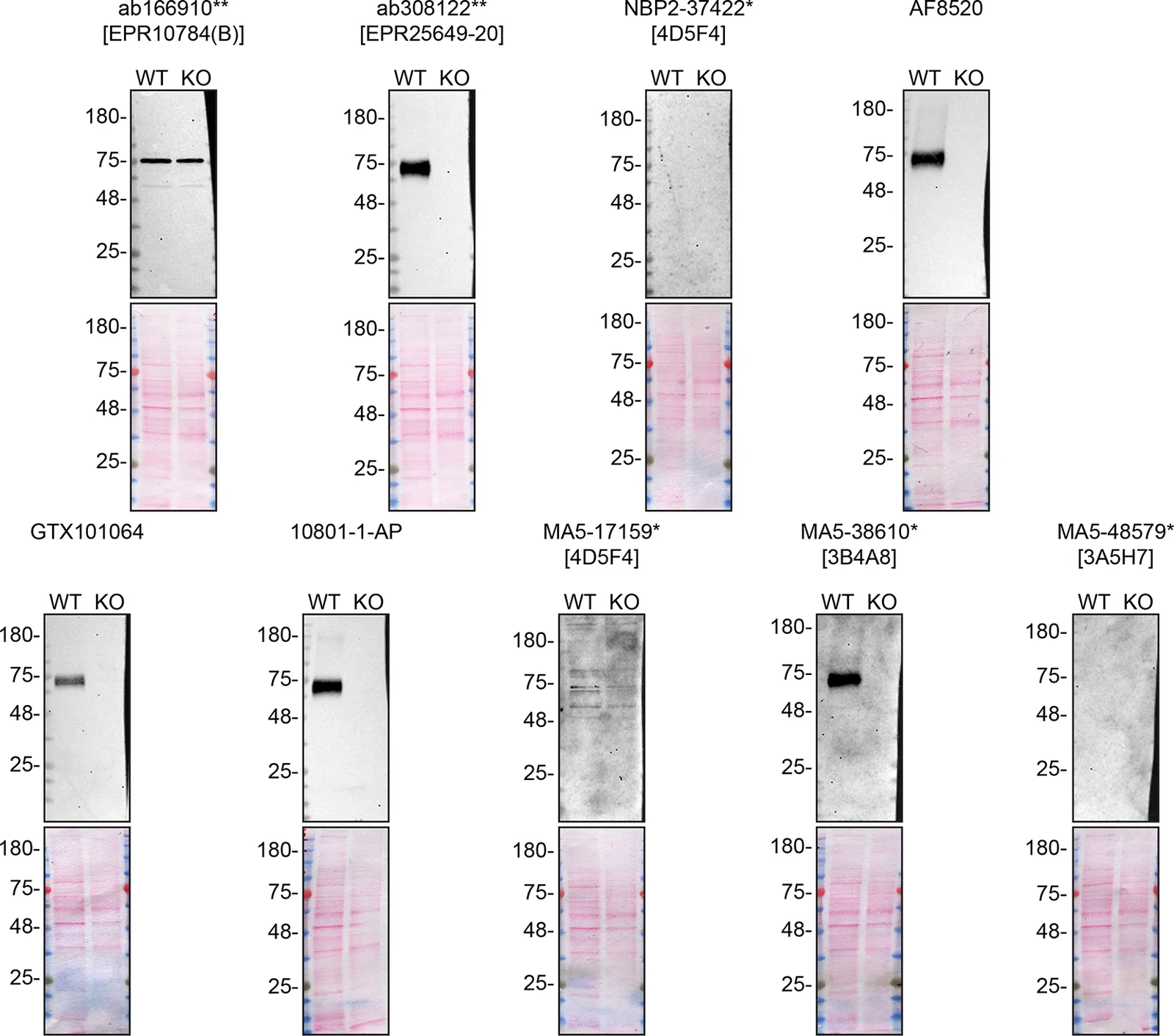
Prosaposin antibody screening by western blot. Culture media from HeLa WT and *PSAP* KO were collected, and 10 μg of protein were processed for western blot with the indicated Prosaposin antibodies. The Ponceau stained transfers of each blot are presented to show equal loading of WT and KO samples and protein transfer efficiency from the acrylamide gels to the nitrocellulose membrane. Antibody dilutions were chosen according to the recommendations of the antibody supplier. Antibody dilutions used: ab166910** at 1/1000, ab308122** at 1/1000, NBP2–37422* at 1/200, AF8520 at 1/1000, GTX101064 at 1/500, 10801–1-AP at 1/1000, MA5–17159* at 1/200, MA5–38610* at 1/200, and MA5–48579* at 1/200. Predicted band size: 58.1 kDa. ** = recombinant antibody, * = monoclonal antibody.

Prosaposin functions both as a secreted protein and intracellularly within the lysosome. Our aim was to detect both pools of Prosaposin. We screened all nine Prosaposin antibodies in western blot using HeLa WT and *PSAP* KO cells, analyzing both concentrated culture medium and cellular protein lysates. Samples were ran on SDS-PAGE, transferred onto nitrocellulose membranes, and then probed in parallel with the nine Prosaposin antibodies. While several antibodies detected Prosaposin in the culture medium ( Figure 1), intracellular Prosaposin was barely detectable in cell lysates (data not shown). This may be due to proteolytic cleavage of full-length Prosaposin within the cell, likely disrupting the epitopes required for antibody recognition.

**Figure 2.**
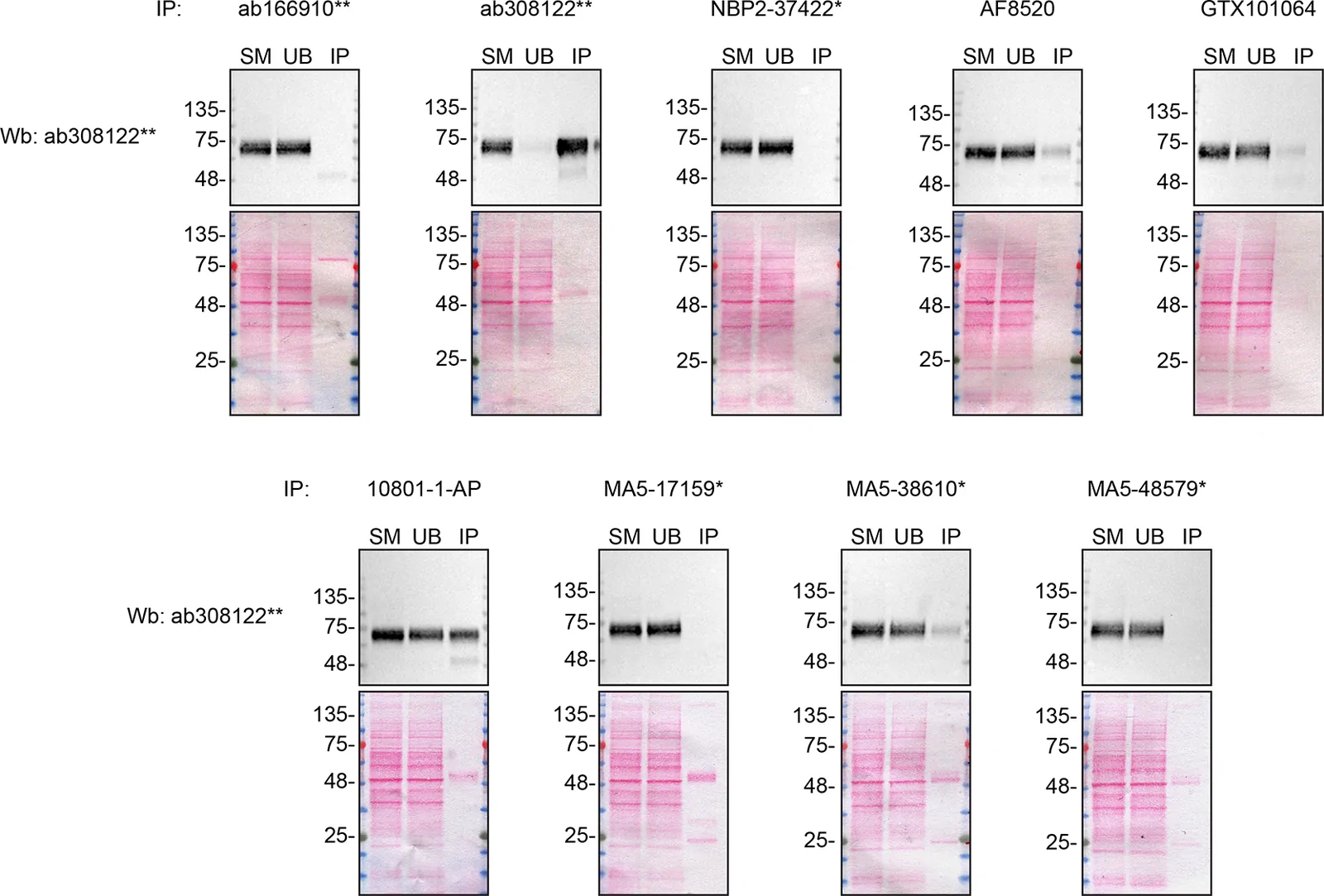
Prosaposin antibody screening by immunoprecipitation. Culture medium was collected from HeLa WT, and immunoprecipitation was performed for 1 h using 0.3 mg of protein and 2.0 μg of the indicated Prosaposin antibodies pre-coupled to Dynabeads protein A or protein G. Samples were washed and processed for western blot with the anti-Prosaposin ab308122** diluted at 1/1000. The Ponceau stained transfers of each blot are shown. SM = 10% starting material; UB = 10% unbound fraction; IP = immunoprecipitate. ** = recombinant antibody, * = monoclonal antibody.

We then assessed the capability of all nine antibodies to capture Prosaposin from HeLa WT culture medium using the immunoprecipitation technique, followed by western blot analysis. For the immunoblot step, a specific Prosaposin antibody identified previously (refer to Figure 1) was selected. Equal amounts of the starting material (SM) and the unbound fractions (UB), as well as the whole immunoprecipitate (IP) eluates were separated by SDS-PAGE ( Figure 2).

In conclusion, we have screened nine Prosaposin commercial antibodies by western blot and immunoprecipitation by comparing the signal produced by the antibodies in human HeLa WT and *PSAP* KO cells. To assist users in interpreting antibody performanyce, Table 4 outlines various scenarios in which antibodies may perform in these applications. ^10^ High-quality and renewable antibodies that successfully detect Prosaposin was identified in both applications. Researchers who wish to study Prosaposin in a different species are encouraged to select high-quality antibodies, based on the results of this study, and investigate the predicted species reactivity of the manufacturer before extending their research.

## Limitations

Inherent limitations are associated with the antibody characterization platform used in this study. Firstly, the YCharOS project focuses on renewable (recombinant and monoclonal) antibodies and does not test all commercially available Prosaposin antibodies. YCharOS partners provide approximately 80% of all renewable antibodies, but some top-cited polyclonal antibodies may not be available through these partners.

Secondly, the YCharOS effort employs a non-biased approach that is agnostic to the protein for which antibodies have been characterized. The aim is to provide objective data on antibody performance without preconceived notions about how antibodies should perform or the molecular weight that should be observed in western blot. As the authors are not experts in Prosaposin, only a brief overview of the protein’s function and its relevance in disease is provided. Prosaposin experts are invited to analyze and interpret observed banding patterns in western blots and subcellular localization in immunofluorescence.

Thirdly, YCharOS experiments are not performed in replicates primarily due to the use of multiple antibodies targeting various epitopes. Once a specific antibody is identified, it validates the protein expression of the intended target in the selected cell line, confirms the lack of protein expression in the KO cell line and supports conclusions regarding the specificity of the other antibodies. Moreover, the same antibody clones are donated by 2–3 manufacturers (cross-licensed antibodies), effectively serving as replicates and enabling the validation of test reproducibility. All experiments are performed using master mixes, and meticulous attention is paid to sample preparation and experimental execution. In IF, the use of two different concentrations serves to evaluate antibody specificity and can aid in assessing assay reliability. In instances where antibodies yield no signal, a repeat experiment is conducted following titration. Additionally, our independent data is performed subsequently to the antibody manufacturers internal validation process, therefore making our characterization process a repeat.

Lastly, as comprehensive and standardized procedures are respected, any conclusions remain confined to the experimental conditions and cell line used for this study. The use of a single cell type for evaluating antibody performance poses as a limitation, as factors such as target protein abundance significantly impact results. Additionally, the use of cancer cell lines containing gene mutations poses a potential challenge, as these mutations may be within the epitope coding sequence or other regions of the gene responsible for the intended target. Such alterations can impact the binding affinity of antibodies. This represents an inherent limitation of any approach that employs cancer cell lines.

## Method

The standardized protocols used to carry out this KO cell line-based antibody characterization platform was established and approved by a collaborative group of academics, industry researchers and antibody manufacturers. The detailed materials and step-by-step protocols used to characterize antibodies in western blot, immunoprecipitation and immunofluorescence are openly available on Protocols.io ( protocols.io/view/a-consensus-platform-for-antibody-characterization ). ^6^ Brief descriptions of the experimental setup used to carry out this study can be found below.

### Cell lines and antibodies

Cell lines used and primary antibodies tested in this study are listed in Table 1 and 2, respectively. To ensure that the cell lines and antibodies are cited properly and can be easily identified, we have included their corresponding Research Resource Identifiers, or RRID. ^11^ ^,^ ^12^

Peroxidase-conjugated goat anti-rabbit and anti-mouse antibodies are (Thermo Fisher Scientific, cat. Number 65–6120 and 62–6520). Peroxidase-conjugated Protein A for IP detection is from Cell Signaling Technology, cat. Number 12291 ( Table 3).

### Antibody screening by western blot

HeLa WT and *PSAP* KO cells were washed 3x with PBS 1x and starved for ~36 hrs. Culture media were collected and centrifuged for 10 min at 500 x *g* to eliminate cells and larger contaminants, then for 10 min at 4500 x *g* to eliminate smaller contaminants. Culture media were then concentrated by centrifuging at 4000 x *g* for 30 min using the Amicon Ultra-15 Centrifugal Filter Units with a membrane NMWL of 10 kDa (MilliporeSigma cat. Number UFC901024). Culture media were finally supplemented with 1x protease inhibitor cocktail mix (MilliporeSigma, cat. Number P8340). BLUelf prestained protein ladder (GeneDireX, cat. Number PM008–0500) was used.

Western blots were performed with precast midi 4–20% Tris-Glycine polyacrylamide gels (Thermo Fisher Scientific, cat. Number WXP42012BOX) ran with Tris/Glycine/SDS buffer (Bio-Rad, cat. Number 1610772), loaded in Laemmli loading sample buffer (Thermo Fisher Scientific, cat. Number AAJ61337AD) and transferred on nitrocellulose membranes. Proteins on the blots were visualized with Ponceau S staining (Thermo Fisher Scientific, cat. Number BP103–10) which is scanned to show together with individual western blot. Blots were blocked with 5% milk for 1 hr, and antibodies were incubated O/N at 4 °C with 5% milk in TBS with 0,1% Tween 20 (TBST) (Cell Signalling Technology, cat. Number 9997). Following three washes with TBST, the peroxidase conjugated secondary antibody was incubated at a dilution of ~0.2 μg/ml in TBST with 5% milk for 1 hr at room temperature followed by three washes with TBST. Membranes were incubated with Pierce ECL (Thermo Fisher Scientific, cat. Number 32106) or Clarity Western ECL Substrate (Bio-Rad, cat. Number 1705061) prior to detection with the iBright™ CL1500 Imaging System (Thermo Fisher Scientific, cat. Number A44240).

### Antibody screening by immunoprecipitation

Antibody-bead conjugates were prepared by adding 2 μg of antibody to 500 μl of Pierce IP Lysis Buffer (25 mM Tris-HCl pH 7.4, 150 mM NaCl, 1 mM EDTA, 1% NP-40 and 5% glycerol) from Thermo Fisher Scientific, cat. Number 87788 in a microcentrifuge tube, together with 30 μl of Dynabeads protein A- (for rabbit antibodies) or protein G- (for rat and sheep antibodies) (Thermo Fisher Scientific, cat. Number 10002D and 10004D, respectively). Tubes were rocked for ~1 h at 4 °C followed by two washes to remove unbound antibodies.

Culture media from HeLa WT were collected as described in the western section above. 0.38 mL aliquots at 0.8 mg/mL of culture medium were incubated with an antibody-bead conjugate for ~1 h at 4 °C. The unbound fractions were collected, and beads were subsequently washed three times with 1.0 mL of IP buffer and processed for SDS-PAGE and western blot on precast midi 4–20% Tris-Glycine polyacrylamide gels. Protein A:HRP was used as a secondary detection system at a concentration of 0.5 μg/ml.

## Data Availability

Zenodo: Dataset for the Prosaposin antibody screening study < https://doi.org/10.5281/zenodo.18445428>. Data are available under the terms of the Creative Commons Attribution 4.0 International license (CC-BY 4.0).
