# Supplementary material for: A guide to selecting high-performing antibodies for Prosaposin (UniProt ID: P07602) for use in western blot and immunoprecipitation
Source: F1000Res. 2026 Jul 4;15:1065. [Version 1] doi: 10.12688/f1000research.177779.1 (PMC13615352; doi:10.12688/f1000research.177779.1)
Supplement: Peer review history

# Reviewer report: Reviewer response for version 1

**DOI:** 10.5256/f1000research.196064.r521239

**Published:** 2026-09-25

**Authors:** Carolyn Jones

**Affiliations:** 1 University of Leicester, Leicester, England, UK

The article (A guide to selecting high-performing antibodies for Prosaposin (UniProt ID: P07602) for use in western blot and immunoprecipitation) is generally clear, concise and easy to follow.  The rationale is well-described, and the results are laid out in a sensible way.  It is clear that the authors are testing a range of commercial antibodies, using a WT and a KO cell line.  They are not recommending any particular antibody but are providing a resource for researchers to use in their own decision making.

 There are a few typographical errors, which seem to stem from the article being copy-pasted from another similar publication, so are easily fixed.  There are several mentions of immunofluorescence in the article, but this technique is not done here. 

1. In Table 4, the title and legend are: “Table 4. Illustrations to assess antibody performance in all western blot, immunoprecipitation and immunofluorescence.”  Since IF is not done here, it should be removed from both.
2. The Limitations section states: “In IF, the use of two different concentrations serves to evaluate antibody specificity…” IF is not done here, so this is confusing and should be removed.
3. Paragraph 1 of the Method section says: “The detailed materials and step-by-step protocols used to characterize antibodies in western blot, immunoprecipitation and immunofluorescence are openly available on Protocols.io”.  While this is correct, again the reference to IF is not needed.

Another small error with Table 4 is that the image headings are the wrong way round (i.e. Western Blot is over the image of IP, and vice versa).

 Additionally, I would suggest that the Suppliers’ recommended dilution ranges are shown on Table 2, along with the dilutions used.  This would make it clearer to compare (e.g. have the lowest recommended dilutions been used, or an average etc).

Are sufficient details of methods and materials provided to allow replication by others?

Yes

Is the rationale for creating the dataset(s) clearly described?

Yes

Are the datasets clearly presented in a useable and accessible format?

Yes

Are the protocols appropriate and is the work technically sound?

Yes

Reviewer Expertise:

Genomics, cancer research, respiratory, quality management

I confirm that I have read this submission and believe that I have an appropriate level of expertise to confirm that it is of an acceptable scientific standard.

# Reviewer report: Reviewer response for version 1

**DOI:** 10.5256/f1000research.196064.r507868

**Published:** 2026-08-21

**Authors:** Deborah Moshinsky

**Affiliations:** 1 Institute for Protein Innovation, Boston, Massachusetts, USA

Prosaposin functions as a regulator of lysosomal enzymatic activity and as a neuroprotective agent.  It is active in lysosomes and as a secreted protein.  This study tested 9 Prosaposin commercially available antibodies for their ability to detect this protein in media from HeLa cells and corresponding isogenic Prosaposin knockouts (KO).  The experimental conditions are clearly described and the protocols are standardized.  Results showed the expected detection of a band of approximately Prosaposin's molecular weight in wild type cells but not in KOs for some antibodies, and various other staining patterns for others.  

 The authors also tested the antibodies for their ability to immunoprecipitate (IP) the target using a standardized protocol.  2 antibodies resulted in successful IP, matching the general pattern of staining expected. 

 This study should assist researchers in selecting Prosaposin antibodies that are likely to work for their experimental use. Although every experimental result is dependent upon the exact conditions used, the study should serve as an appropriate rough guide.

Are sufficient details of methods and materials provided to allow replication by others?

Yes

Is the rationale for creating the dataset(s) clearly described?

Yes

Are the datasets clearly presented in a useable and accessible format?

Yes

Are the protocols appropriate and is the work technically sound?

Yes

Reviewer Expertise:

Antibody Characterization and Validation

I confirm that I have read this submission and believe that I have an appropriate level of expertise to confirm that it is of an acceptable scientific standard.
